# PNSS: An online plant name service system

**DOI:** 10.3897/BDJ.13.e142973

**Published:** 2025-03-31

**Authors:** Qiu Jinshui, Zhang Jianwen, Jin Tao, Zhuang Huifu

**Affiliations:** 1 National Wild Plant Germplasm Resource Center, Kunming Institute of Botany, Chinese Academy of Sciences, Kunming, China National Wild Plant Germplasm Resource Center, Kunming Institute of Botany, Chinese Academy of Sciences Kunming China; 2 Science and Technology Information Center, Kunming Institute of Botany, Chinese Academy of Sciences, Kunming, China Science and Technology Information Center, Kunming Institute of Botany, Chinese Academy of Sciences Kunming China; 3 State Key Laboratory of Phytochemistry and Natural Medicines, Kunming Institute of Botany, Chinese Academy of Sciences, Kunming, China State Key Laboratory of Phytochemistry and Natural Medicines, Kunming Institute of Botany, Chinese Academy of Sciences Kunming China

**Keywords:** plant name, data integration, plant name retrieval, plant name matching, plant name search, plant name parsing

## Abstract

Biodiversity plays a vital role in human survival and development. Consequently, the protection of biodiversity has become a global concern. Biological names serve as biological identifiers and the use of correct biological names helps promote biodiversity conservation research. At present, there are numerous taxonomic databases and software tools available worldwide for processing plant names. However, these resources are scattered across various database websites or personal computers. Users must invest a significant amount of time collecting these resources and expend substantial effort to learn, use and maintain them, consequently leading to high user learning and usage costs. Here, we propose a solution to address the above problem. We collected mainstream and freely available taxonomic datasets from around the world, integrated them into an extensive taxonomic dataset and subsequently mapped the data in this summary database to Solr search engine. Then, based on these taxonomic datasets, we designed database, algorithms and system, developed the system and finally established an online plant name service system (PNSS). The PNSS not only integrates the mainstream taxonomic datasets, but also offers free plant name retrieval, matching, search, parsing and application programming interface (API) services to help biologists conduct more effective research on biodiversity conservation.

## Introduction

The 15^th^ meeting of the Conference of the Parties to the Convention on Biological Diversity (CBD COP15) was held in Kunming, Yunnan, China, in 2021. This was the first global conference convened by the United Nations with the theme of “Ecological Civilization”. In 2022, the second phase of CBD COP15 was held in Montreal, Canada, and the Kunming–Montreal Global Biodiversity Framework was adopted. It is evident that biodiversity conservation has become a hot issue requiring global cooperation. Biological names serve as identifiers of various organisms on Earth. They are the link between multimodal data (e.g. species descriptions, colour images, specimen records and video data) associated with organisms (Penev et al. 2016, Schoch et al. 2020). Collecting, cleaning and integrating biological name data are fundamental, labour-intensive, yet essential tasks in biological research and biodiversity conservation (Qian and Jin 2016, Wagner and Palmer 2016, Jin and Qian 2022, Jin and Qian 2023). Cleaning biodiversity data contributes to research on biodiversity and promotes global biodiversity conservation. For example, in research fields such as statistics and analysis of species richness, prediction of species distribution and data mining of biodiversity, using the correct biological names can help researchers reduce taxonomic bias and obtain accurate and high-quality biodiversity data. Therefore, an accurate and appropriate biological name database is of great help to zoologists, botanists, microbiologists and ecologists (Pyle 2016).

Fortunately, there are many free and open taxonomic databases worldwide, such as Global Biodiversity Information Facility Backbone Taxonomy (GBIF Secretariat 2023), Catalogue of Life (Bánki et al. 2024), World Flora Online (WFO 2023), Plants of the World Online (POWO 2024), Leipzig Catalogue of Vascular Plants (Freiberg et al. 2020) and Catalogue of Life China (The Biodiversity Committee of Chinese Academy of Sciences 2024). Although most taxonomic databases provide plant name retrieval and matching services, only a few databases (e.g. Global Biodiversity Information Facility and Catalogue of Life) provide plant name parsing and Application Programming Interface (API) services and they basically do not provide plant name search services. Therefore, currently, users are inconvenienced in the following ways: (1) Users need to visit database websites to retrieve the required plant names. Sometimes, users must visit multiple database websites to find these names, increasing the time cost; (2) Due to the inconsistent rules governing plant name API services across various database websites, users have to implement different plant name APIs for different database websites, adding to the technical complexity; (3) As some database websites do not yet offer plant name matching, parsing and search services (Qiu et al. 2022), when users require such services, they generally need to use third-party software tools such as TaxonFinder (TaxonFinder 2023), TaxonMatch (Rees 2014) or others developed in R (Alvarez and Luebert 2018, Brown et al. 2023, Zhang and Qian 2023). However, using multiple software tools to process plant names requires users to spend more time and costs learning the relevant knowledge of each software tool, thereby increasing their workload; (4) In addition, TaxonFinder is still a popular software tool in the field of biodiversity informatics, usually used to search for plant names from text content. However, since it was developed nearly a decade ago, its design is actually incomplete. As it only considers whether the genus and specificEpithet are correct individually, but cannot correctly determine the overall correctness of the composition of genera and specificEpithet. The plant name “Camellia henryi” is selected here as an example, while the individual words for the genus and specificEpithet are correct, but in reality, this is not a correct plant name. However, TaxonFinder considers this plant name to be correct.

**Figure 1.**
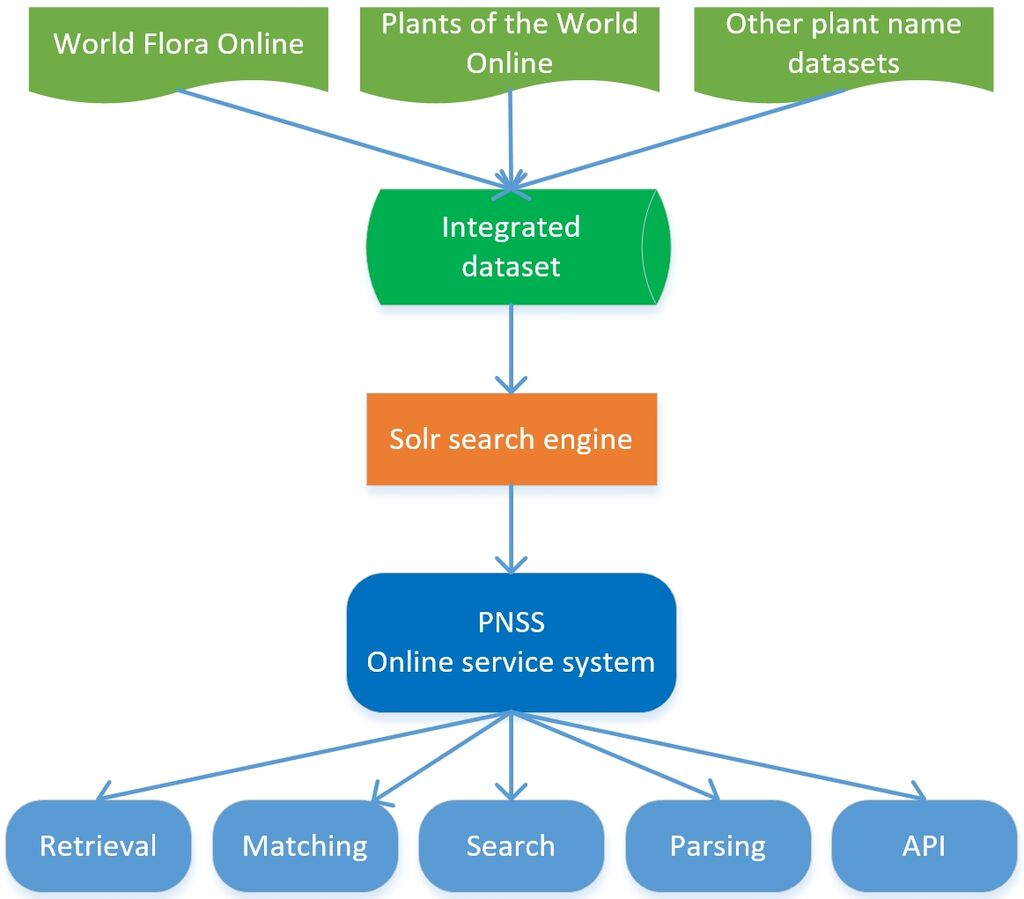
The overall technical approach of PNSS.

Here, we propose a solution to address the above problems. We collect all the mainstream taxonomic datasets in the world, including World Flora Online, Plants of the World Online and other taxonomic datasets. Subsequently, we extract the core data from these taxonomic datasets and add them to a database table, which we refer to as the plant name summary table, thereby allowing us to construct an extensive integrated taxonomic dataset containing most of the plant names in the world. To enhance the efficiency of retrieving these names, we map this extensive plant name summary table of plant names to the Solr (Solr 2025) search engine to create an index for all the integrated plant names. Then, we develop a free online service system (PNSS) to offer users plant name retrieval, matching, search, parsing and API services. When users utilise various services of this online system, they can either opt to select all integrated taxonomic datasets by default or choose only a specific taxonomic dataset they desire. Therefore, this online service system integrates multiple taxonomic datasets and offers various plant name services. Our overall technical approach is illustrated in Fig. 1.

## Materials and methods

### Data collection and processing

Plant names are mainly stored in multiple databases around the world, including comprehensive databases such as Global Biodiversity Information Facility (GBIF) Backbone Taxonomy and Catalogue of Life (COL), as well as professional databases in the field of plant science, such as World Flora Online (WFO), Plants of the World Online (POWO), Leipzig Catalogue of Vascular Plants (LCVP) and some national databases such as Catalogue of Life China (COLChina). We collected mainstream, free and open downloadable taxonomic datasets from around the world. Thus far, the valid taxonomic datasets that we collected and integrated are shown in Table 1. We then imported the collected data into a Structured Query Language (SQL) server database, extracted the core data from these taxonomic data about plants and summarised them in a database table. We refer to this integrated extensive database table as the “plant name summary table”. Finally, we mapped the data in the plant name summary table to the Solr search engine to create an index table that provides a name search service for the online service system. The creation of this index table ensures that the online service system can efficiently and rapidly search and match plant names in the massive data.

### System design

**Figure 2.**
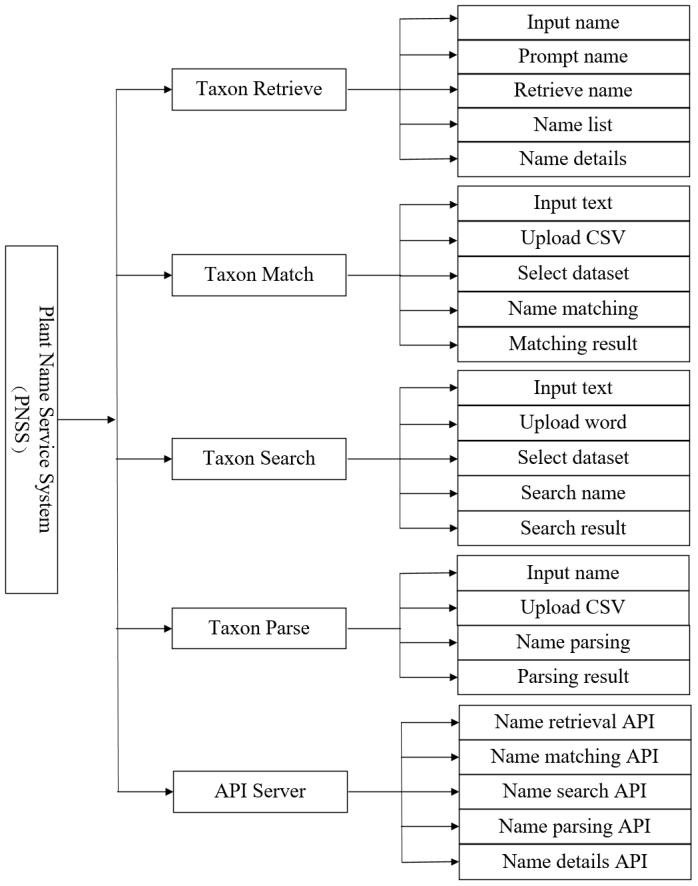
The functions and modules of PNSS. From left to right, it is respectively "system - modules - functions".

The main purpose of designing this online service system is to assist users in accessing multiple services, such as plant name retrieval, matching, search, parsing and API services, on a single website. When utilising these services, users have the option to choose any of the integrated taxonomic datasets. Additionally, we offer multiple service modes for some service items. For example, plant name matching services can be provided by inputting text or uploading CSV. Throughout the design process, we consistently adhered to a straightforward, easy-to-use system design. Fig. 2 shows the overall functional module design of the online service system.

### Database design

**Figure 3.**
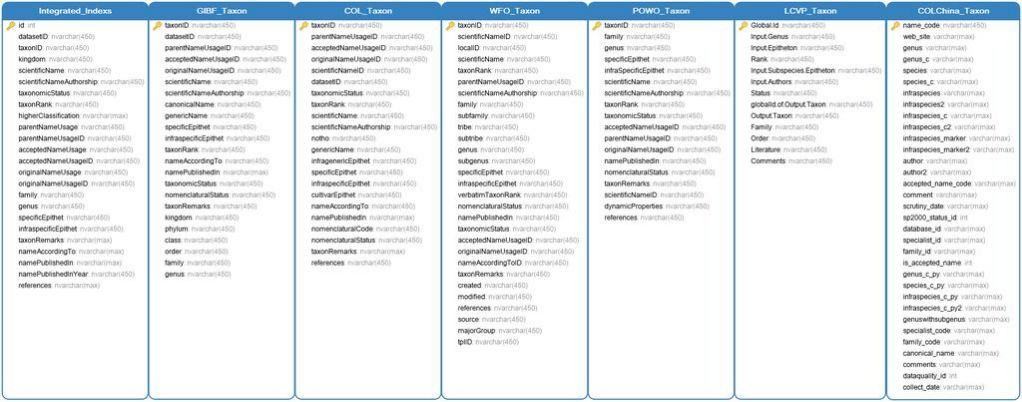
Database table designs for taxonomic datasets and the plant name summary table. The GBIF_Taxon table is used to store the taxonomic data of the GBIF Backbone Taxonomy, the COL_Taxon table is used to store the taxonomic data of the Catalogue of Life, the WFO_Taxon table is used to store the taxonomic data of World Flora Online, the POWO_Taxon table is used to store the taxonomic data of Plants of the World Online, the LCVP_Taxon table is used to store the taxonomic data of The Leipzig Catalogue of Vascular Plants, the COLChina_Taxon table is used to store the taxonomic data of the Catalogue of Life China and the Integrated_Indexs table is used to store the plant names summarised from the six taxonomic datasets mentioned above, which is the plant name summary table.

The database software we used is SQL Server 2016 Express. To facilitate the management of each taxonomic dataset while maintaining their independence, we created a separate database for each dataset to store the original data. Since the datasets we integrated are all mainstream taxonomic datasets, we consider them to be authoritative and accurate. Therefore, we did not modify or delete the original data of these taxonomic datasets. Instead, we designed the corresponding database table for each dataset based on its attributes. To summarise the core data of various taxonomic datasets, we designed a plant name summary table with reference to the Darwin Core standard to store the core data of each taxonomic dataset (Wieczorek et al. 2012). The designs of the database tables for taxonomic datasets and the plant name summary table are shown in Fig. 3.

### Algorithm design

Before developing the plant name service system, we designed the relevant algorithms that the system needs to use, mainly including plant name preprocessing algorithms, plant name retrieval algorithms, plant name matching algorithms, plant name search algorithms and plant name parsing algorithms.

#### Preprocessing algorithms

The first step is preprocessing the plant names as it is a necessary step. The main function of preprocessing is to make the writing of plant names more standardised and unified, including the handling of special characters and spaces in names, such as converting uppercase special characters to lowercase special characters, in order to facilitate subsequent plant name processing. For example, when dealing with spaces, first insert a space after the character that needs to retain a space (such as the character "."), then convert multiple consecutive spaces into one space (ensuring that there is only one space after each character that needs to retain a space) and finally remove the space before a specific character (such as the character ")"), this ensuring that the writing of plant names is more standardised.

#### Retrieval algorithms

The plant name retrieval algorithms are used to retrieve qualified target results from integrated taxonomic datasets based on the plant name entered by user and return the target results. When retrieving preprocessed plant names, the plant names are first split into n items by space and then the data that meets these items are fuzzily retrieved from the database. Then, the retrieved plant names are filtered, based on other filtering conditions such as the namePublishedInYear, taxonRank, taxonomicStatus, dataSet and whether to retrieve vernacularName. The search results are then sorted in ascending order according to the length of the plant name. Finally, the search results are grouped and statistically analysed, based on the namePublishedInYear, taxonRank, taxonomicStatus, dataSet and the search results are returned.

#### Matching algorithms

The plant name matching algorithms are used to find the most similar plant name from the integrated taxonomic datasets, based on the plant name entered by the user, then to calculate the similarity coefficient of the two plant names and finally to return the results. When matching preprocessed plant names, the first step is to perform an exact match. If the match is complete, stop matching and return the exact match result; otherwise, perform a fuzzy matching of one distance, that is, search for plant names in the database that differ by one character from the given plant name (possibly one more character, one less character or one different character). If such a plant name exists, stop matching and return to the fuzzy matching result; otherwise, perform a fuzzy matching of two distances, that is, search for plant names in the database that differ by two characters from the given plant name. If such a plant name exists, stop matching and return to the fuzzy matching result; otherwise, determine whether the given plant name contains scientificNameAuthorship. If it does, delete the scientificNameAuthorship and repeat the matching process. Finally, calculate the similarity between each matching result and the given plant name and sort the matching results in descending order based on the similarity.

#### Search algorithms

The plant name search algorithms are used to find the correct plant names from the text content entered by the user, to tag these correct plant names and then to return these correct plant names and tag information. The simple method of searching for plant names from the text is to traverse each plant name in the database to determine if it appears in the text, but this method is inefficient. The search algorithm in this article is to traverse each word in the text one by one to determine whether the word complies with the writing conventions for plant names, such as whether it starts with uppercase or "×". If the word conforms to the writing conventions of plant names, retrieve from the database whether there are plant names starting with the word. If there are no plant names starting with this word in the database, skip the word and repeat the search process for the next word. If there are plant names starting with this word in the database, mark the word as an intermediate result, then read the next word and combine it with the above intermediate result to form a new phrase and then search the database for plant names starting with this phrase. If there are plant names starting with this phrase in the database, mark the phrase as a new intermediate result and then read the next word and combine it with the new intermediate result to form a new phrase. Repeat the search process until no plant name starting with the new phrase can be found in the database. Mark the last intermediate result as a search result, that is, search for a plant name, record the plant name and calculate the starting and ending positions of the plant name in the text. Repeat the above search process until the words in the text are fully traversed.

#### Parsing algorithms

The plant name parsing algorithms are used to parse the plant name entered by user and break it down into multiple parts, including genusOrAbove, specificEpithet, infraspecificEpithet, scientificNameAuthorship and namePublishedInYear etc. and return the above parsing results. The parsing algorithm is mainly designed, based on the rules of the Binomial nomenclature (Turland et al. 2018). Firstly, it determines whether the plant name contains scientificNameAuthorship. If it does not, the plant name is marked as a scientificName. If it does, the plant name is decomposed into a scientificName and scientificNameAuthorship. For a scientificName, if it is a single word, it is marked as a genusOrAbove. If it is multiple words, it is broken down into genusOrAbove, specificEpithet and infraspecificEpithet. For scientificNameAuthorship, it is determined whether the namePublishedInYear is included. If it is not included, it is marked as author name. If it is included, it is broken down into author name and namePublishedInYear and the result of the above decomposition is returned.

### System development

We used Visual Studio Community 2013, an integrated development environment, to develop the online service system. C#, ASP.NET and model–view–controller (MVC) were used on the server side and JavaScript, CSS and bootstrap were used on the web client side. The index data for plant names were stored in the Solr search engine and the original data for plant names were stored in the SQL server database. We developed the online service system by using the above software tools and development techniques. The server receives user request information from the web client. The request information may include plant name data or plant name files as well as some processing parameters. Next, the server preprocesses these plant name data and other information, then calls different Solr retrieval functions or database retrieval functions depending on the user’s processing needs and finally displays the retrieval results in the user’s web browser, thus fulfilling the user’s service request.

## Results

By collecting various mainstream taxonomic datasets and then storing, extracting, integrating and processing them as well as designing the database, algorithms and system and developing the system, we finally completed all the work for the online PNSS. This system has been officially launched (https://pnss.iflora.cn) and provides online services for researchers in the field of Plants. The homepage of the PNSS is shown in Suppl. material 1: Fig. S1 and mainly offers users plant name retrieval, matching, search, parsing and API services.

### Plant name retrieval service

The PNSS has integrated more than eight million plant names of various types, allowing users to retrieve plant names based on their specific needs.

When a user enters a plant name of interest in the search box, the system automatically suggests plant names similar to the entered characters. The user can either choose a plant name from the suggested list for searching or click the search button after inputting their query. The system then begins searching for the requested plant name and redirects to the search results page once the search is complete. On this page, the search results are presented to the user in a list format. The displayed information includes scientificName, scientificNameAuthorship, acceptedNameUsage and higherClassification etc. The search results data are also summarised and categorised based on different dimensions such as taxonRank, taxonomicStatus, namePublishedInYear and dataset. Users can filter the information they desire using these different dimensions. For example, if a user searches for “Acer integrum”, the search results page, as shown in Suppl. material 1: Fig. S2, allows the user to click on a link for a specific scientificName to open and view the original detailed information associated with this scientificName.

### Plant name matching service

The plant name matching service assists users in checking the correctness of the plant names they submit. The PNSS compares the user-submitted plant names with the selected taxonomic dataset or all taxonomic datasets by default. The plant name submitted by the user that matches exactly with the plant name in the database will be marked as "Match" and plant names with one or two differenced characters will be marked as "Recorrect". Users can refer to the correct plant name to correct it. If the matching is a vernacularName, it will be marked as a vernacularName and other plant names will be marked as "Unmatch". The plant name matching service allows users to submit plant names online in two ways.

**Figure 4.**
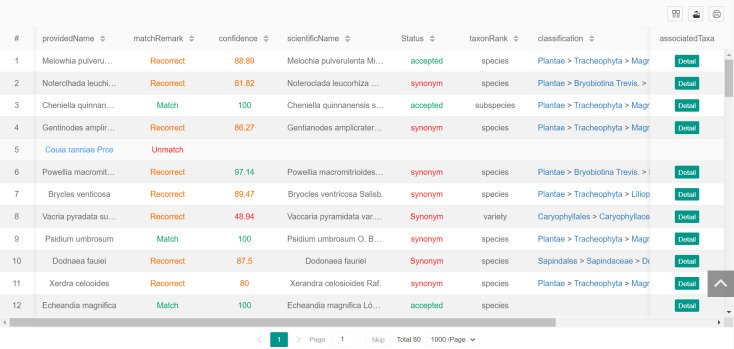
The results page of plant name matching.

The first way to submit plant names is to enter the plant name(s) that the user desires to match in the text box. Each line in the text box can accommodate one plant name and a total of 1000 lines can be entered, that is, the system supports the matching of up to 1000 plant names in a single submission. After the system receives the plant names submitted by the user, it begins the matching process. After the matching is completed, the system displays the results on the matching result page, as shown in Fig. 4. The user can view the results online or click the “Detail” button to view the original detailed information of the matched plant names.

The second way to submit plant names is by uploading a comma-separated values (CSV) file. The PNSS provides users with a CSV template file (https://pnss.iflora.cn/Resource/Files/TaxonMatchTemplate.csv), users can download the template file, enter the plant names they wish to match into it and then upload it to the PNSS. Once receiving the CSV file containing the plant names, the PNSS first reads the plant names in the CSV file and then executes the matching operation. After the matching is completed, the PNSS writes the matching result and relevant information for each plant name into a new Excel file and returns its address to the user, who can then download this file.

### Plant name search service

The plant names search service helps users find all plant names from text content. Users can choose a specific taxonomic dataset for reference or use all the integrated taxonomic datasets by default. This service supports users in submitting text content online in two ways.

The first way of submitting text content is to enter text content in the text box and then click the “Start Search” button. Once the system receives the text content submitted by the user, it searches for the plant names. After the search is complete, the system displays the results on the search results page. To facilitate users in quickly identifying the plant names found during the search, the background colour of these names is displayed in green, as shown in Suppl. material 1: Fig. S3. Users can also download the search result file, which include scientificName, startIndex, endIndex, taxonRank and taxonomicStatus etc.

The second way to submit text content is to upload a Microsoft Word document containing text content. The user inputs the text content into a Word document and then uploads it to the PNSS. After receiving the Word document, the PNSS first reads the text content and then starts to search for the plant names. After the search is completed, the PNSS sets the background colour of each found plant name to green, writes this information into a new Word document and returns the address of this new Word document to the user, who can then download the file.

### Plant name parsing service

The method of plant name parsing service is similar to that of matching service. Users can enter the plant name to be parsed in the text box or upload a CSV file containing the plant name to be parsed. After the parsing is completed, users can view the parsing results and also download the parsing result file for plant names. The parsing results mainly include parseRemark, scientificName, scientificNameAuthorship and namePublishedInYear etc., as shown in Suppl. material 1: Fig. S4.

### Plant name API service

The PNSS not only provides users with online services such as plant name retrieval, matching, search and parsing, but also offers users plant name API services. Currently, the PNSS provides users with five types of plant name API services, namely, the plant name retrieval API (https://pnss.iflora.cn/Api/Retrieval), the plant name matching API (https://pnss.iflora.cn/Api/Match), the plant name search API (https://pnss.iflora.cn/Api/ Search), the plant name parsing API (https://pnss.iflora.cn/Api/Parse) and the plant name details API (https://pnss.iflora.cn/Api/Detail). The specific instructions for using these different types of plant name API services can be obtained by visiting https://pnss.iflora.cn/Home/API.

### Comparison with GBIF

GBIF is the world's largest online sharing platform for biodiversity data, providing users with online services such as plant name retrieval, matching and parsing, but does not offer online plant name search services. PNSS not only provides users with different plant name retrieval, matching and parsing services, but also provides users with online plant name search services.

In terms of name retrieval, GBIF not only returns some basic information about the plant name, but also returns occurrences with images and georeferenced records related to the species, which is very helpful for users to understand where the plant has appeared and what it looks like. PNSS focuses more on which datasets have included this plant name and how it is described in each dataset. In addition, we have extracted and supplemented other data information of this name, based on its association.

In terms of name matching, we randomly selected 6000 plant names and divided them into three categories: correct plant names, randomly wrong one character for each word in the name and randomly wrong two characters for each word in the name (shown in Suppl. material 2). Then, we used a computer programme to process the above three types of names into names for testing. The name matching test results showed that 2701 plant names were successfully recalled on GBIF, including 1795 correct plant names, 757 randomly incorrect plant names of one character and 149 randomly incorrect plant names of two characters. Interestingly, 5642 plant names were successfully recalled on PNSS, including 2000 correct plant names, 1985 randomly incorrect plant names of one character and 1657 randomly incorrect plant names of two characters. Therefore, the matching recall rate of PNSS is higher than that of GBIF. The test results are shown in Suppl. materials 3, 4.

In terms of name parsing, we randomly selected 6000 plant names with a name status of "accepted" for testing (shown in Suppl. material 5). The name-parsing test results showed that PNSS can resolve all plant names, but GBIF has 23 plant names that cannot be resolved, mainly because these plant names contain characters such as "_x" or "×". In addition, PNSS also extracts the namePublishedInYear included in the name and provides users with associated plant names, which not only helps users parse plant names, but also provides other reference information for plant names. The test results are shown in Suppl. materials 6, 7.

## Conclusion

We implemented the designed technical solution, completed the collection, storage, extraction, integration and processing of the world’s major taxonomic datasets, developed an online service PNSS and conducted extensive tests on this system, confirming its effectiveness. By using this PNSS, users can retrieve most of the plant name information in the world without the need to visit the websites of multiple taxonomic databases. Additionally, users can obtain plant name matching, search and parsing services on the same website, eliminating the need to install multiple software tools for processing plant names and sparing users the responsibility of maintaining and updating any of the taxonomic datasets themselves. In addition, our PNSS provides APIs for the above services. Consequently, users do not need to visit our website, but only need to call our APIs to access various plant name services. By integrating taxonomic datasets and services, our PNSS can effectively assist Botanists in processing plant names with less time, effort and money, allowing them to channel more of their efforts into specialised research areas such as biodiversity conservation.

Admittedly, there is room for improvement. Although we integrated most of the plant names in the world, due to the vast number of global taxonomic datasets, we have not yet integrated 100% of these names. However, we will gradually integrate other taxonomic datasets. Due to technical, personnel, time and funding reasons, our integrated taxonomic data about plants cannot be updated in real time. However, we will make an effort to update all integrated taxonomic datasets to their latest versions on an annual basis. In addition, we will continuously enhance our website, constantly improve the functional modules of the website and regularly optimise our algorithms for retrieval, matching, search and parsing. We strive to provide free, visually appealing, feature-rich and more efficient and accurate plant name processing services for biologists, thereby contributing to biodiversity conservation.

## Supplementary Material

F3A6E78E-45DA-5B38-9D59-769B518998D9 10.3897/BDJ.13.e142973.suppl1 Supplementary material 1 Some usage examples of the PNSS Data type Figures Brief description Some usage examples of the PNSS. File: oo_1288763.docx https://binary.pensoft.net/file/1288763 Qiu Jinshui, Zhang Jianwen, Jin Tao, Zhuang Huifu

0D26CB56-885E-58DF-96F5-811C1B8A9931 10.3897/BDJ.13.e142973.suppl2 Supplementary material 2 The test data of plant name matching-20240307 Data type Excel File: oo_1288764.xlsx https://binary.pensoft.net/file/1288764 Qiu Jinshui, Zhang Jianwen, Jin Tao, Zhuang Huifu

3891C4AD-5BC9-5651-B722-31425D3A9CEE 10.3897/BDJ.13.e142973.suppl3 Supplementary material 3 Test results of using GBIF to match plant names Data type CSV File: oo_1184309.csv https://binary.pensoft.net/file/1184309 Qiu Jinshui, Zhang Jianwen, Jin Tao, Zhuang Huifu

3655F52D-88D6-5DE1-B8D8-5E5C22534CA5 10.3897/BDJ.13.e142973.suppl4 Supplementary material 4 Test results of using PNSS to match plant names Data type Excel File: oo_1184311.xlsx https://binary.pensoft.net/file/1184311 Qiu Jinshui, Zhang Jianwen, Jin Tao, Zhuang Huifu

6D2479F5-D5AE-576A-B425-9FE413CFA754 10.3897/BDJ.13.e142973.suppl5 Supplementary material 5 The test data of plant name parsing-20240319 Data type Excel File: oo_1288766.xlsx https://binary.pensoft.net/file/1288766 Qiu Jinshui, Zhang Jianwen, Jin Tao, Zhuang Huifu

612C8CC5-311E-5C2A-A046-8A354B011A3E 10.3897/BDJ.13.e142973.suppl6 Supplementary material 6 Test results of using GBIF to parse plant names Data type CSV File: oo_1184312.csv https://binary.pensoft.net/file/1184312 Qiu Jinshui, Zhang Jianwen, Jin Tao, Zhuang Huifu

D6A796CB-A9FF-5FBD-8C0D-A5B55B550020 10.3897/BDJ.13.e142973.suppl7 Supplementary material 7 Test results of using PNSS to parse plant names Data type Excel File: oo_1184313.xlsx https://binary.pensoft.net/file/1184313 Qiu Jinshui, Zhang Jianwen, Jin Tao, Zhuang Huifu

## Figures and Tables

**Table 1.** The integrated taxonomic datasets.

#	DatasetID	Dataset Name	Publication Date	Number of Plant Names	Integrated Date
1	COL	Catalogue of Life	19/12/2024	1,565,194	04/01/2025
2	COLChina	Catalogue of Life China	22/05/2024	178,019	18/06/2024
3	GBIF	GBIF Backbone Taxonomy	28/08/2023	2,241,809	01/02/2024
4	LCVP	Leipzig Catalogue of Vascular Plants	08/11/2022	1,337,891	01/02/2024
5	POWO	Plants of the World Online	12/06/2024	1,431,677	18/06/2024
6	WFO	World Flora Online	22/12/2023	1,576,137	18/06/2024
