# Supplementary material for: PNSS: An online plant name service system
Source: Biodivers Data J. 2025 Mar 31;13:e142973. doi: 10.3897/BDJ.13.e142973 (PMC11976303; doi:10.3897/BDJ.13.e142973)
Supplement: Supplementary material 1 — Some usage examples of the PNSS [file bdj-13-e142973-s001.docx]

**Supplementary material**

Title: PNSS: An online plant name service system

Authors: Qiu Jinshui, Zhang Jianwen, Jin Tao, Zhuang Huifu


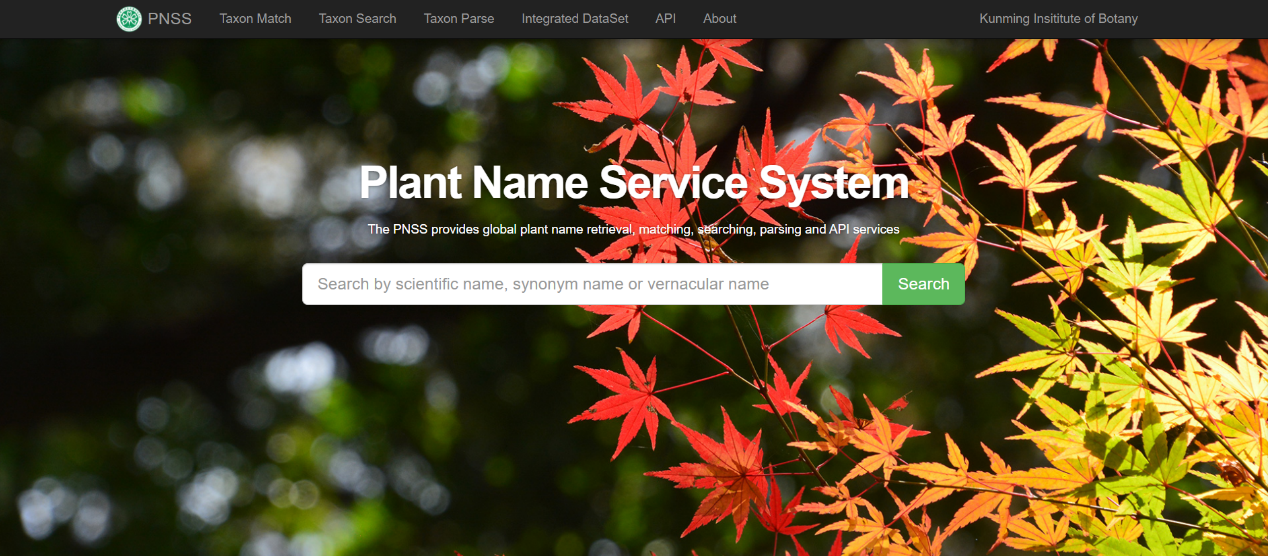


Fig. S1. Homepage of the PNSS website.


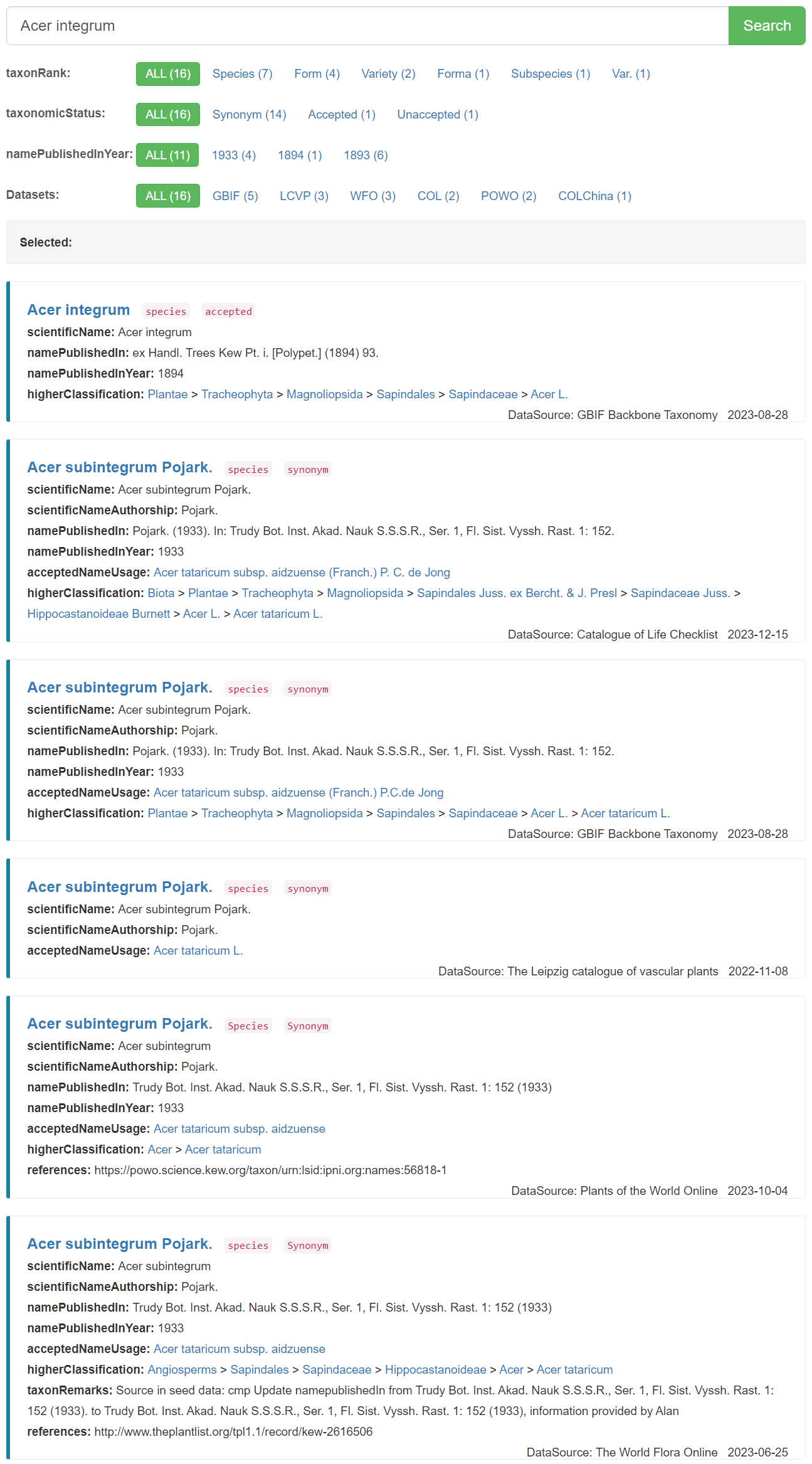


Fig. S2. The search result page of *Acer integrum.*


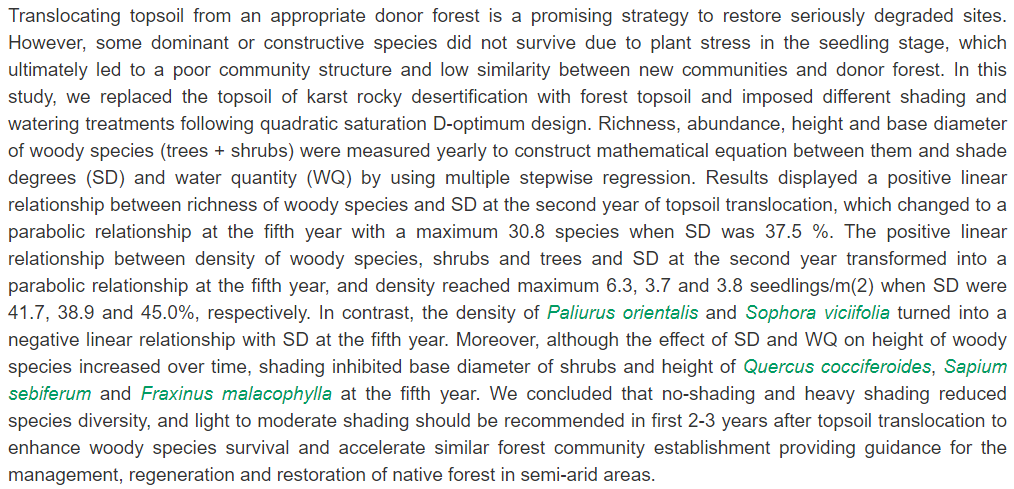


Fig. S3. The results page of plant name search.


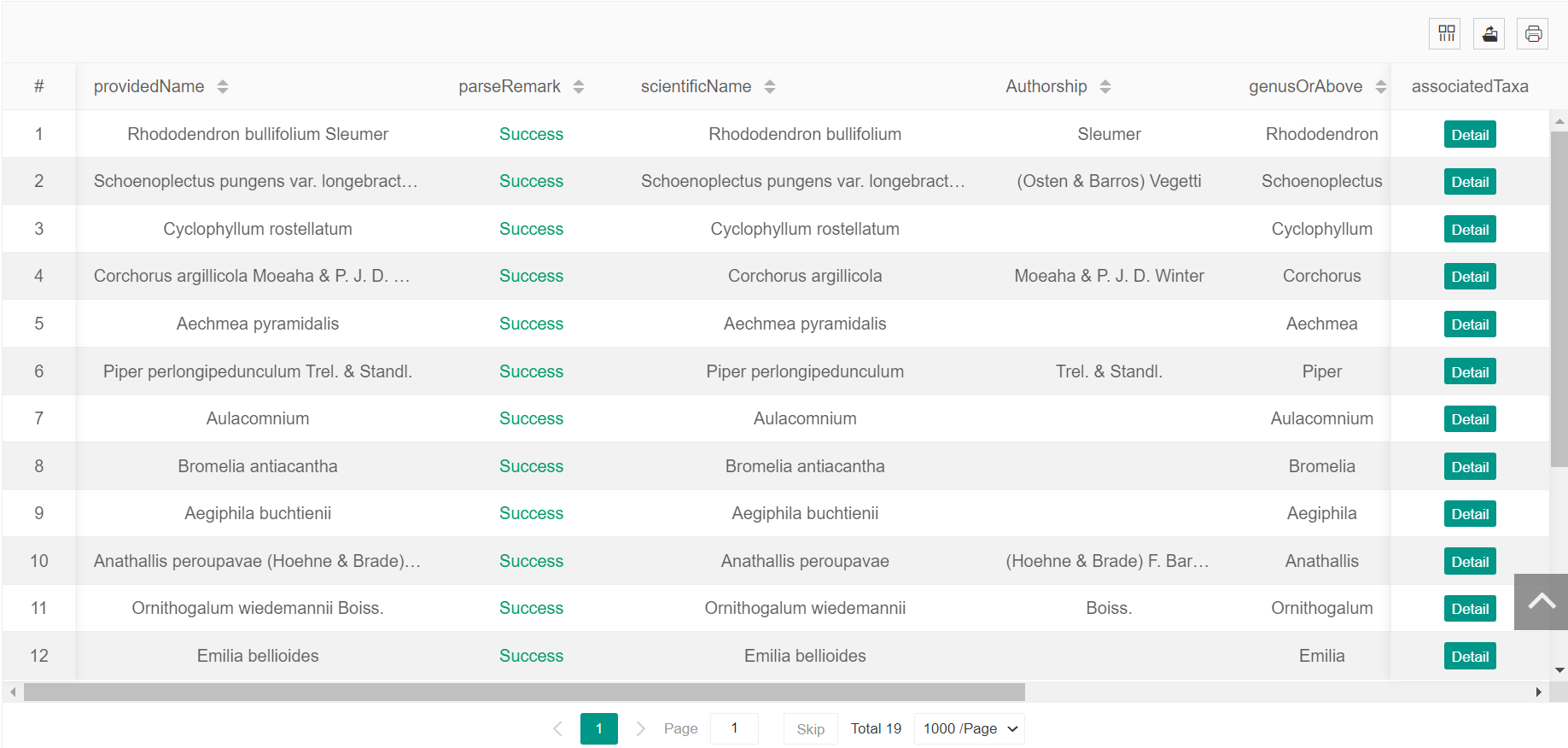


Fig. S4. The parsing result of plant names.
